# Animal models of hyperandrogenism and ovarian morphology changes as features of polycystic ovary syndrome: a systematic review

**DOI:** 10.1186/s12958-017-0231-z

**Published:** 2017-02-10

**Authors:** Larissa Paixão, Ramon B. Ramos, Anita Lavarda, Debora M. Morsh, Poli Mara Spritzer

**Affiliations:** 1 Gynecological Endocrinology Unit, Division of Endocrinology, Hospital de Clínicas de Porto Alegre, Rua Ramiro Barcelos, 2350, 90035 003 Porto Alegre, RS Brazil; 2 Department of Physiology, Laboratory of Molecular Endocrinology, Federal University of Rio Grande do Sul, Porto Alegre, Brazil

**Keywords:** Animal models, Ovary, PCOS, Androgens, Estrogens, Transgenic animals, Rodents, Non-human primates

## Abstract

Polycystic ovary syndrome (PCOS) is a common endocrine disorder, affecting 9–18% of women in reproductive age that causes hyperandrogenism and infertility due to dysfunctional follicular maturation and anovulation. The etiology of PCOS is still poorly known, and information from experimental animal models may help improve current understanding of the mechanisms of PCOS initiation and development. Therefore, we conducted a systematic review of currently available methods for simulation of PCOS in experimental models, focusing on two main endocrine traits: ovarian morphology changes and circulating levels of sex hormones and gonadotropins.

We searched the MEDLINE database for articles in English or Spanish published until October 2016. Of 933 studies identified, 39 were included in the systematic review. One study compared interventions with androgens versus estrogens, 18 used androgen-induced stimulation, 9 used estrogens or drugs with estrogen action, including endocrine disruptors, to induce PCOS-like models, and 12 used miscellaneous interventions. Broad differences were found among the studies concerning hormonal interventions, animal species, and developmental stage at the time of the experiments, and most models resulted in ovarian morphology changes, mainly increases in the number of cystic and antral follicles and decreases in the corpus luteum. Hyperandrogenism was produced by using androgens and other drugs as the stimulatory agent. However, studies using drugs with estrogenic effect did not observe changes in circulating androgens.

In conclusion, medium- or long-term testosterone administration in the pre- and postnatal periods performed best for induction of a PCOS-like phenotype, in rhesus macaque and rat models respectively. In rats, postnatal exposure to androgens results in reprogramming of the hypothalamic-pituitary-ovarian-axis. Thus, comparisons between different intervention models may be useful to define the timing of reproductive PCOS phenotypes in experimental animal models.

## Background

Polycystic ovary syndrome (PCOS) is one of the most common endocrine conditions, affecting 9–18% of women of reproductive age, depending on diagnostic criteria [1]. PCOS is characterized by at least two of the three following criteria: clinical or biochemical hyperandrogenism, oligo/anovulation, and polycystic ovaries [2]. Clinically, these manifestations are associated with reduced fertility, due to dysfunctional follicular maturation and consequent anovulation, and hyperandrogenism, causing acne and hirsutism [3]. Both chronic anovulation and androgen excess are linked to disturbed folliculogenesis, that is expressed by multiple cystic follicles between 2 and 9 mm and increased ovarian volume in women with PCOS [4].

Clinical PCOS may have its onset before or during puberty [5]. Clinical studies have suggested that girls with PCOS exhibit increased gonadotropin releasing hormone (GnRH) pulse frequency, leading to excess luteinizing hormone (LH) secretion. This causes premature acquisition of LH receptor expression by growing ovarian follicles at excessively early stages, leading to increased ovarian androgen production [6, 7] and, probably, to the arrested antral follicle development of PCOS [8]. Indeed, this follicular arrest is consistent with the polycystic ovary morphology found on ultrasound examination in human PCOS [2]. Moreover, PCOS-affected ovaries exhibit an increase in the number of growing preantral and antral follicles, which leads to antrum expansion, increased granulosa cell degeneration, and development of cystic follicles, with thin granulosa cell walls and a thicker surrounding layer of theca cells [8].

Since Stein and Leventhal first described PCOS in the mid-1930s [9], the search began to identify the etiologic mechanisms associated with its development. Yet, despite its prevalence and health impact, the etiology of PCOS remains poorly understood. Even whether reproductive hormone abnormalities are primary or secondary remains enigmatic. Hypotheses for the origins of this pathology include hormonal imbalances, genetic abnormalities, and lifestyle and environmental factors [3]. Due to logistic and ethical limitations on human experimentation, several animal models to simulate PCOS have been developed in recent decades. These experimental models can improve understanding of the pathophysiology of PCOS and have the potential to support development of innovative and curative treatments.

Within this context, the aim of this paper was to systematically review the available rodent and non-human primate animal models of PCOS-like conditions and summarize ovarian function and hormonal disturbances linked to reproductive damage, as well as discuss the advantages and possible limitations of applying these models to human PCOS.

## Methods

### Search strategy and study selection

We performed a systematic review in accordance with the PRISMA guidelines. Briefly, we searched the MEDLINE database, via PubMed, for literature published in English and Spanish until October 2016. The search strategy consisted of the following Medical Subject Headings (MeSH): “Animal Model” OR “Animal Models” OR “Model, Animal” OR “Laboratory Animal Models” OR “Animal Model, Laboratory” OR “Animal Models, Laboratory” OR “Laboratory Animal Model” OR “Model, Laboratory Animal” OR “Models, Laboratory Animal” OR “Experimental Animal Models” OR “Animal Model, Experimental” OR “Animal Models, Experimental” OR “Experimental Animal Model” OR “Model, Experimental Animal” OR “Models, Experimental Animal” OR Rodents OR Rodentias OR Rodent OR Rat OR Rattus OR “Rattus norvegicus” OR “Rats, Norway” OR “Rats, Laboratory” OR “Laboratory Rat” OR “Laboratory Rats” OR “Rat, Laboratory” OR Mus OR Mouse OR “Mus musculus” OR “Mice, House” OR “House Mice” OR “Mouse, House” OR “House Mouse” OR “Mus domesticus” OR “domesticus, Mus” OR “Mus musculus domesticus” OR “domesticus, Mus musculus” OR “musculus domesticus, Mus” OR “Mice, Laboratory” OR “Laboratory Mice” OR “Mouse, Laboratory” OR “Laboratory Mouse” OR “Mouse, Swiss” OR “Swiss Mouse” OR “Swiss Mice” OR “Mice, Swiss” OR AND “Ovary Syndrome, Polycystic” OR “Syndrome, Polycystic Ovary” OR “Stein-Leventhal Syndrome” OR “Stein Leventhal Syndrome” OR “Syndrome, Stein-Leventhal” OR “Sclerocystic Ovarian Degeneration” OR “Ovarian Degeneration, Sclerocystic” OR “Sclerocystic Ovary Syndrome” OR “Polycystic Ovarian Syndrome” OR “Ovarian Syndrome, Polycystic” OR “Polycystic Ovary Syndrome 1” OR “Sclerocystic Ovaries” OR “Ovary, Sclerocystic” OR “Sclerocystic Ovary”.

The selection criteria were as follows: experimental studies of interventions in female rats, mice, guinea pigs, or non-human primates aiming to induce PCOS-like reproductive characteristics, namely, follicles with cystic appearance and altered serum testosterone levels. We also performed a hand search of the reference lists of full-text articles. Studies were excluded from the analysis if the outcome was not induction of PCOS symptoms in experimental animals and if reproductive outcomes were nor described.

### Data extraction

Two investigators (LP and RBR) independently reviewed the titles and abstracts and selected articles for full-text review. Disagreements were resolved by a third reviewer (PMS) or by consensus discussion. The selected articles were independently read in full to confirm eligibility and extract data. The information extracted from each individual study was as follows: name of first author, publication year, animal species, number of animals, age at start and at intervention time, intervention type, experimental period, and three variables of interest: serum levels of sex hormones, serum levels of gonadotropin, and ovarian morphology.

## Results

### Study selection

**Fig. 1:**
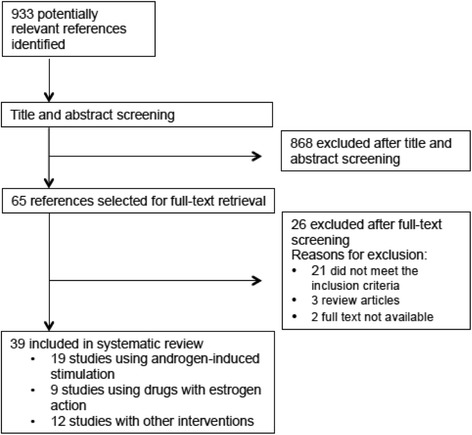
Animal models using androgen-induced stimulation

A detailed flow diagram of the study selection process is shown in Fig. 1. The primary search identified 933 articles. After title and abstract screening, 65 potentially eligible studies were retrieved for full-text review. Of these, 26 were excluded: 21 did not meet the inclusion criteria; 3 were narrative reviews; and 2 were very old publications that could not be retrieved. Therefore, 39 studies were included in the systematic review: 19 animal models using androgen-induced stimulation [10–28]; 9 which employed estrogens and estrogen-like drugs to induce a PCOS-like syndrome [27, 29–36]; and 12 using other interventions [37–48].

Table 1 summarizes the characteristics of the studies included in the systematic review that used androgens as the stimulus to induce a PCOS-like phenotype. Most of the studies used rodents (3 studied mice, 11 used rats) and 5 used monkeys as the experimental model. Table 1 also shows that androgen induction was applied at different periods in the reported studies: short-term (up to 30 days of androgen induction) [12, 19, 20, 26, 27]; medium-term (30 to 90 days) [10, 11, 13–16, 21, 25]; and long-term (>90 days) [17, 22–24]. In addition, interventions were applied to animals at distinct developmental stages, as described below.

**Table 1:** Animal models using androgen-induced stimulation

Author, year [reference]	Animal	N	Intervention and duration	Studied variables	Results
Intervention period: Fetal					
Tyndall et al. 2012 [11]	Wistar rat	--	(Fetal and postnatal) Testosterone propionate 25–90 days	sexual hormones gonadotropin ovarian morphology	↑T = E2 ↓ FSH ↑ cystic follicles No CL
Wu et al. 2010 [10]	Sprague–Dawley rat	45	(PA) Testosterone + DHT 70 days	sexual hormones gonadotropin ovarian morphology	↑ T ↑E2 ↑LH = FSH No cysts ↓CL
Caldwell et al. 2014 [13]	Mouse	--	PA: DHT Postnatal: DHT/DHEA/Letrozole During 90 days	sexual hormones gonadotropin ovarian morphology	=T = E2 =FSH = LH ↑ cystic follicles ↑ atretic
Yan et al. 2013 [12]	Sprague Dawley rat	44	(PA) DHT 30 days	sexual hormones ovarian morphology	=T = E2 ↑ cystic follicles ↑ atretic follicles
Abbott et al. 1998 [25]	Rhesus macaque	21	Testosterone propionate 15–88 days	sexual hormones gonadotropin ovarian morphology	↑T ↑LH ↓FSH ↑multiple follicles >1 mm
Intervention period: Postnatal					
Ota et al. 1983 [17]	Wistar–Imamichi rat	55-77	Testosterone propionate Single dose Observed for 200 days	sexual hormones gonadotropin ovarian morphology	↑T ↓E2 ↑LH ↑FSH ↑ cystic follicles No CL
Zhai et al. 2012 [15]	Sprague–Dawley rat	30	Andronate/ Andronate + HFD 60 days	sexual hormones gonadotropin ovarian morphology	↑T ↑ cystic follicles =FSH ↑LH
Misugi et al. 2006 [18]	Wistar rat	30	DHEA (not reported)	sexual hormones ovarian morphology	↑T ↑ cystic follicles ↑atretic follicles
Van Houten et al. 2012 [14]	C57/bL6 female mouse	--	DHT 90 dias	sexual hormones	↑ DHT
Manneras et al. 2007 [16]	Wistar rat	--	DHT 75 days	sexual hormones ovarian morphology	=T = E2 ↑ cystic follicles
Paixão et al. 2016 [28]	Wistar rat	20	eCG + DHEA	sexual hormones ovarian morphology	↑T ↑ cystic follicles
Ongaro et al. 2015 [26]	Sprague–Dawley rats	35	Testosterone propionate Single dose During 30 and 60 days	sexual hormones ovarian morphology	=T = E2 ↑ cystic follicles (60 days-old)
Marcondes et al. 2015 [27]	Wistar rats	10	Testosterone propionate Single dose	sexual hormones gonadotropin ovarian morphology	↑T ↑LH = FSH ↑ cystic follicles No CL
Intervention period: Pubertal					
Familiari et al. 1985 [19]	Mouse	--	DHEA 20 days	sexual hormones ovarian morphology	↑T ↑ cystic follicles
Knudsen et al. 1975 [20]	Holtzman rat	12/16	DHEA 20 days	sexual hormones, gonadotropin ovarian morphology	=T = E2 =FSH = LH No cysts
Intervention period: Adult					
Tang et al. 2012 [21]	Female Rhesus macaque	6	Testosterone propionate + hCG 60 days	sexual hormones gonadotropin ovarian morphology	↑T = E2 ↑LH = FSH ↑ cystic follicles
McGee et al. 2014 [22]	Rhesus macaque	12	Testosterone + HFD 72 months	sexual hormones gonadotropin ovarian morphology	↑T ↓LH amplitude ↑antral follicles
Billiar et al. 1985 [24]	Rhesus macaque	--	Androstenedione 10 months	sexual hormones gonadotropin ovarian morphology	↑T =FSH = LH ↑ atretic follicles No cysts
Faiman et al. 1988 [23]	Rhesus macaque	25	Testosterone 13–16 months	sexual hormones ovarian morphology	=T No cysts No CL =antral follicles

*T* testosterone, *E2* estradiol, *LH* luteinizing hormone, *FSH* follicle-stimulating hormone, *CL* corpus luteum, *PA* prenatal androgenization, *DHT* dihydrotestosterone, *DHEA* dehydroepiandrosterone, *HFD* high-fat diet; ↑: increase; ↓ decrease; = equal; --: not available

Five studies described prenatal interventions and observed the offspring at different periods of development [10–13, 25]. Three of these studies used rats as the experimental model [10–12], one utilized female mice [13], and another used rhesus macaques [25]. Wu et al. (2010) [10] and Yan et al. (2013) [12] used the Sprague–Dawley rat strain for medium- and short-term induction, respectively. Wu et al. (2010) [10] administered testosterone during gestation and dihydrotestosterone (DHT) through 70 days after birth and found elevated testosterone levels, but no ovarian changes. Yan et al. (2013) [12] administered DHT during gestation and found only cystic and atretic follicles. Caldwell et al. (2014) [13] administered DHT during gestation plus DHT or dehydroepiandrosterone (DHEA) as postnatal treatment in female mice, and observed cystic and atretic follicles only. Similar findings were reported by Tyndall et al. (2012) [11], who administered pre- and postnatal treatment with testosterone propionate for 25–90 days and observed cystic follicles as the only change. Finally, Abbott et al. (1998) [25] administered testosterone propionate for 15–88 days, starting at various gestational ages, and found consistent reproductive features similar to clinical PCOS in female rhesus macaque offspring, such as increased serum testosterone and LH levels, lower follicle stimulating hormone (FSH) levels, and ovaries containing multiples cystic follicles.

Eight studies described postnatal interventions and observed offspring at different periods of development. Seven of these studies used female rats [15–18, 26, 27] and one used female mice [14]. While most studies used testosterone or DHT, only two studies administered DHEA to induce ovarian dysfunction [18, 28]. Misugi et al. (2006) [18] observed both cystic/atretic follicles and high testosterone levels in response to DHEA stimulation. Paixão et al. (2015) used a single equine chorionic gonadotropin (eCG) dose to induce initial follicular recruitment in prepubertal rats and added DHEA to generate ovarian dysfunction. These animals presented greater testosterone levels and higher number of, and larger-sized, primary and secondary follicles as compared with the control group [28].

Ota et al. (1983) [17] also found cystic follicles and elevated testosterone levels after administration of testosterone propionate injections to Wistar rats. Ongaro et al. (2015) [26] administered a single dose of testosterone propionate to 5-day-old female Sprague–Dawley rats, and found no differences in testosterone or estradiol levels at 30 and 60 days. Cystic follicles were observed only in 60-day-old animals. Marcondes et al. (2015) [27] administered a single dose of testosterone propionate to 0-to-2-day-old female Wistar rats. Increased testosterone levels, cystic follicles, and absence of corpora lutea were found at 90–94 days of age. Manneras et al. (2007) [16] administered DHT to the same rat strain and found cystic follicles, but no testosterone changes. Unlike Ota et al. (1983) [17], who conducted a long-term intervention, the study by Manneras et al. (2007) [16] had a medium-term observation period. Both Zhai et al. (2012) [15] and van Houten et al. (2012) [14] also conducted experiments of intermediate duration. These two studies found cystic follicles and increased androgen levels, despite using different animals and different types of androgens as intervention. While van Houten et al. (2012) [14] observed female mice under DHT treatment, Zhai et al. (2012) [15] studied female Sprague–Dawley rats receiving testosterone plus a high-fat diet.

Two studies described postnatal interventions and observed the offspring at different periods of development [19, 20]. In these studies, DHEA was administered for 20 days to female mice [19] and to female rats [20]. One study did not find any changes [20], whereas the other reported cystic follicles and increased testosterone levels [19].

The four available studies of adult animals were performed on non-human primates [21–24]. Tang et al. (2012) [21] observed cystic follicles and increased testosterone levels after medium-term treatment with testosterone propionate plus hCG injection. The other three studies used long-term treatment with androgens [22–24]. McGee et al. (2014) [22] found antral follicles and high levels of testosterone after treatment with testosterone plus a high-fat diet. One study of androstenedione intervention [24] found increased testosterone levels and atretic follicles but no cystic or antral follicles. Faiman et al. (1988) [23], using testosterone as the intervention, did not observe any changes in hormone levels or ovarian morphology.

### Animal models using drugs with estrogenic effects

Table 2 shows the characteristics of studies that used estrogen or drugs with estrogenic effects to induce a PCOS-like phenotype in experimental animals. These studies used acute estrogen treatment, lasting hours to days [27, 29–31, 34, 35], and short-term interventions, lasting 2 to 30 days [32, 33, 36]. All models used rodents, at distinct developmental stages: eight studies used rats and one used guinea pigs.

**Table 2:** Studies using drugs with estrogenic effects

Author, year [reference]	Animal	N	Intervention	Studied variables	Results
Intervention period: Postnatal and puberty					
Cruz et al. 2012 [29]	Sprague–Dawley rats	30	Estradiol valerate Single dose	Sexual hormones Gonadotropin Ovary morphology	↑ E2 ↓ androstenedione = LH ↑ atretic follicles ↓ corpora lutea ↑ cystic follicles
Schulster et al. 1984 [30]	Wistar rats (pubertal)	65	Estradiol valerate Single dose	Sexual hormones Gonadotropin Ovary morphology	↓ E2 ↓ LH ↑ cystic follicles
Brawer et al. 1986 [31]	Wistar rats (pubertal)	50	Estradiol valerate Single dose	Gonadotropin Ovary morphology	↓ LH ↓ FSH ↑ cystic follicles ↑ atretic follicles No CL
Fernández et al. 2010 [32]	Sprague–Dawley rats	30	Bisphenol A 10 days	Sex hormones Ovary morphology	↑E2 ↑ T ↑ cystic follicles ↑ atretic follicles ↓ CL
Marcondes et al. 2015 [27]	Wistar rats	10	Estradiol Benzoate Single dose	Sexual hormones Gonadotropin Ovarian morphology	=T = LH = FSH ↑ cystic follicles No CL
Intervention Period: Adulthood					
Hemmings et al. 1983 [35]	Wistar rats	32	Estradiol valerate Single dose	Sexual hormones Gonadotropin Ovary morphology	= T ↓ LH ↑ cystic follicles
Quandt et al. 1993 [34]	Guinea pigs	32	Estradiol-17β 2 days	Sexual hormones Ovary morphology	↑ E2 = androstenedione ↑ cystic follicles ↑ atretic follicles
Zangeneh et al. 2011 [33]	Wistar rats	48	Estradiol valerate Single dose + cold stress simultaneously	Sex hormones Gonadotropin Ovary morphology	↑ E2 = LH = FSH ↑ cystic follicles
McCarthy & Brawer, 1990 [36]	Wistar rats	58	E2 pellets 50 days	Sexual hormones Gonadotropin Ovary morphology	= E2 ↑ cystic follicles No CL

*T* testosterone, *E2* estradiol, *LH* luteinizing hormone, *FSH* follicle-stimulating hormone, *CL* corpus luteum; ↑: increase; ↓ decrease; = equal

Four studies described the use of drugs with estrogen action during the postnatal and pubertal periods, at different time points of observation. One [29] used the Sprague–Dawley rat strain, while three [27, 30, 31] used female Wistar rats. In all four studies, the intervention consisted of a single dose of estradiol valerate and cystic follicles were observed after treatment. Regarding sex hormone levels, Cruz et al. (2012) [29] and Marcondes et al. (2015) [27] failed to demonstratehigh androgen levels, while the other two studies [30, 31] did not report blood androgen concentrations.

Four studies described interventions with estradiol valerate or 17β-estradiol in adult animals, with different treatment periods. Three of the studies used female Wistar rats [33, 35, 36], while one [34] used guinea pigs as the model organism.

Fernández et al. (2010) [32] exposed female Sprague–Dawley rats to bisphenol A (BPA) for 10 days. This study was able to show the main reproductive outcomes expected for an animal model of PCOS, such as the presence of cystic and atretic follicles alongside elevated testosterone levels.

Hemmings et al. (1983) [35] administered a single dose of estradiol valerate, whereas Quandt et al. (1993) [34] used estradiol injections over 2 days. Although these studies used different rodent models, both detected the presence of cystic follicles and unchanged androgen levels.

Two of the studies [33, 36] used distinct interventions and durations in the same animal strain. Zangeneh et al. (2011) [33] administered a single dose of estradiol valerate plus cold stress induction, while McCarthy and Brawer (1990) [36] used implanted estradiol pellets and an observation period of 50 consecutive days. Both studies found cystic follicles in the ovaries, but did not measure blood androgen levels.

### Animal models using miscellaneous interventions

Table 3 presents the characteristics of studies that used interventions other than androgen and estrogen treatment to induce PCOS-like phenotypes. These miscellaneous interventions included transgenic animals [37–39] or specific strains [46], drugs that stimulate gonadotropin secretion, such as letrozole [40, 48] and human chorionic gonadotropin (hCG) [44], stressful conditions known to affect the hypothalamic-pituitary-ovarian axis, such as chronic cold stress [42] and light exposure [41, 47] and other drugs (D-galactose and valproic acid) [43, 45]. All of these studies used rodent models: 4 used mice [37, 39, 43, 46] and 8 had rats as the model organism [38, 40–42, 44, 45, 47, 48]. Intervention durations were varied, with short-term, medium-term, and long-term treatment all represented. The interventions were applied to animals at distinct developmental stages, as described below.

**Table 3:** Animal models of miscellaneous interventions

Author, year [reference]	Animal	N	Intervention	Variables studied	Results
Intervention period: Embryonic					
Risma et al. 1997 [37]	Mouse	20	transgenic (bLHβ-CTP)	Sexual hormones Gonadotropin Ovary morphology	↑ E2_,_ ↑ T ↑ LH ↑ cystic follicles
Shi et al. 2009 [38]	Rat	18	transgenic (JCR:LA-cp)	Sexual hormones Ovary morphology	↑ T = E2 ↑ cystic follicles ↑ atretic follicles
Devin et al. 2007 [39]	Mouse	39	transgenic (PAI-1)	Sexual hormones Ovary morphology	↑ T ↑ cystic follicles ↓ CL
Intervention period: Puberty					
Kafali et al. 2004 [40]	Rat	34	Letrozole 21 days	Sexual hormones Gonadotropin Ovary morphology	↑ T_,_ ↓E2_,_ ↑ LH, ↑ cystic follicles ↓ CL
Kang et al. 2015 [47]	Sprague–Dawley Rat		light exposition During 112 days	Sexual hormones Ovary morphology	↑ T ↑ cystic follicles ↑ atretic follicles
Intervention period: Adult					
Bernuci et al. 2008 [42]	Rat	17	Chronic cold stress + LC lesion 60 days	Sexual hormones Ovary morphology	↑ T ↑ E2_,_ = LH = FSH ↑ cystic follicles ↓ ovulation
Park and Choi, 2012 [43]	Mouse	15	D-galactose 45 days	Sexual hormone Ovarian morphology	↑ T ↑ cystic follicles
Ota, et al. 1987 [44]	Rat	33	hCG 80 days	Sexual hormones Gonadotropin Ovary morphology	= T ↑ E2 = LH = FSH ↑ cystic follicles
Baldissera, et al. 1991 [41]	Rat	15	Light exposure 74 days	Ovary morphology Gonadotropin	↑ cystic follicles ↓ CL = LH = FSH
Lagace &Nachtigal, 2003 [45]	Rat	22	Valproic acid 30 days	Sexual hormones Ovary morphology	= E2 = T ↑ replace cysts by cystic follicles
Radavelli- Bagatini et al. 2011 [46]	Mouse	45	Genetic	Sexual hormones Gonadotropin Ovary morphology	↓ T ↑ E2 ↓ LH ↓ CL, ↑ replace cysts by cystic follicles, ↑ atretic follicles
Li et al. 2016 [48]	Rat	10	Letrozole	Sexual hormones Gonadotropin Ovary morphology	↑ T, ↓ E2 ↑ LH, ↑ FSH ↑ cystic follicles

*T* testosterone, *E2* estradiol, *LH* luteinizing hormone, *FSH* follicle-stimulating hormone, *CL* corpus luteum; ↑: increase; ↓ decrease; = equal

Three studies described genetic modification at the embryonic development stage, with generation of transgenic animals [37–39]. Two used mice as the model organism [37, 39], while Shi et al. (2009) [38] used female rats. Each study manipulated a different gene. Risma et al. (1997) [37] used recombination of the LHβ gene linked to the coding sequence of the carboxyl-terminal peptide (CTP) of the hCG β-subunit, which promotes increased LH and testosterone secretion as well as polycystic ovaries. Devin et al. (2007) [39] generated a mouse that expresses human plasminogen activator inhibitor-1 (PAI-1) to ascertain whether the increased PAI-1 levels were associated with impaired ovulation. Shi et al. (2009) [38] studied an obese transgenic mouse model with leptin receptor dysfunction, and found increased serum testosterone levels and atretic and cystic follicles.

Two studies described interventions in pubertal animals. Kafali et al. (2004) [40] exposed female mice to three different doses of letrozole, a non-steroidal aromatase inhibitor, whereas Kang at el. (2015) [47] exposed the animals to continuous light for 16 weeks. Both studies found high testosterone levels and cystic follicles.

Seven studies described different interventions in adult animals. Four used rats under short-term [45] or medium-term treatment [41, 42, 44, 48]. Bernuci et al. (2008) [42] demonstrated the role of the locus coeruleus (LC) in cold stress-induced cystic follicles in rats. Ota et al. [44] reported polycystic ovaries in mature rats under hCG stimulation. Baldissera et al. (1991) [41] proposed a simple experimental model for research into the pathophysiology of polycystic ovaries based on continuous exposure to light. Lagace and Nachtigal (2003) [45] exposed animals to valproic acid (VPA), which was associated with the presence of ovarian cysts. Li et al. (2016) used Letrozole and also observed cystic follicles and lower estrogen levels [48]. All of these studies demonstrated an increased number of ovarian cysts, but only two [42] confirmed an increase in testosterone levels. The other three studies reported unchanged testosterone levels [44, 45] or did not measure sex hormone levels [41].

Park and Choi (2012) [43] described a PCOS-like phenotype in a D-galactose-induced aging mouse model with a medium-term observation period. Radavelli-Bagatini et al. (2011) [46] studied a mouse strain known as the New Zealand Obese (NZO), which naturally displays obesity, insulin resistance, and a mild form of diabetes, manifestations similar to those that often occur in women with PCOS. Therefore, in this study, no supplementary stimulus was used to test whether female NZO mice present changes in ovarian morphology that could be associated with metabolic abnormalities. Both studies revealed ovarian morphological changes. Park and Choi [43] reported increased testosterone levels and cystic follicles, whereas Radavelli-Bagatini et al. (2011) [46] demonstrated a high number of cysts and atretic follicles.

## Discussion

Animal models are regarded as valuable tools to investigate pathophysiological processes of human diseases. Indeed, in most cases, because of obvious ethical concerns, some relevant queries cannot be answered by directly studying affected patients. In PCOS, additional complicating issues are its heterogeneous clinical presentation and the fact that the etiology is still not well defined [49]. Therefore, in the present review, we specifically selected studies focusing on two main endocrine traits of PCOS: ovarian morphology changes and circulating levels of sex hormones and gonadotropins.

Reviews about animal models of PCOS have been published previously, and have addressed various features in different animal models of PCOS-like phenotypes [14, 50–54]. However, this is the first systematic review to provide a full list of rodents and non-human primate models generated by distinct interventions, specifically focusing on two main reproductive features present in women with PCOS: hyperandrogenism and polycystic ovaries.

We included 39 experimental studies which used distinct procedures to induce PCOS-like models of ovarian abnormalities and androgen excess, stratified into those using androgens [10–28], estrogens and endocrine disruptors [27, 29–36], or other interventions [37–48]. Overall, there were broad differences among the studies concerning hormonal interventions, animal species, and developmental stage at the time of the experiments. Most resulted in ovarian morphological changes, mainly increases in the number of antral and cystic follicles and decreases in the corpus luteum. However, while a hyperandrogenic status could be induced by using androgens [10–27] and other drugs [37–47] as stimulatory agents, studies using drugs with estrogenic effect did not measure androgen levels or did not observe changes in circulating androgens [27, 29–36]. Therefore, hormonal interventions using androgens seem to promote the most consistent features of a PCOS-like phenotype in animals, as previously suggested by Abbott et al. [49].

Among studies generating a hyperandrogenic state, prenatal exposure of non-human primates to androgens resulted in the most suitable animal model, displaying both metabolic and reproductive characteristics of PCOS [55, 56]. However, these models are expensive and are not readily adaptable to genetic manipulation. In turn, rodent models provide a versatile tool for investigating biological mechanisms associated with the development of PCOS. Among the advantages of using rodent species, their stable genetic backgrounds, ease of handling and maintenance, shorter reproductive lifespan, and short estrous cycles are the most important. However, some aspects that limit the use of rodents for investigation of reproductive features should be taken into consideration. First, rodents are polyovulatory, while women are mono-ovulatory, suggesting that, despite similarities in the hypothalamic-pituitary-ovarian axis, the FSH-dependent follicle selection process in rodents differs from that in women [57, 58]. Second, although the initial stages of follicular growth (from primordial to preantral stage) seem to be comparable between humans and rodents, differences in regulation by intra-ovarian growth cannot be ruled out [59]. Finally, there are marked differences in the timing of onset of folliculogenesis between rodents and women. While the primordial follicle pool and initiation of follicle growth may arise during the later stages of fetal development in humans, these processes occur only during the early postnatal period in rodents [60]. Thus, results obtained from mice and rats may not translate directly to women.

Interestingly, differences in the generation of reproductive phenotypes are observed according to the developmental period in which androgen treatment is started [11]. In this sense, either starting testosterone treatment postnatally [14–18] or administering DHT treatment during the prepubertal period [19, 20] leads to the development of cyst-like follicles. However, postnatal exposure to DHT results in reprogramming of the hypothalamic-pituitary-ovarian-axis [61]. Thus, comparisons between different intervention models may be useful to define the timing of reproductive PCOS phenotypes in experimental animal models. One example is the study of Ota et al. [17], in which, after treatment of 5-day-old female rats with a single dose of testosterone propionate, various reproductive characteristics of PCOS – such as cystic follicles, anovulation, and imbalances in gonadotropins and sex hormones – were later found over a 200-day observation period. These results suggest androgen induction may have indirectly promoted a pathologic elevation of FSH [17] that blocked ovulation and induced cystic formation.

Estrogens and drugs with estrogenic effects have been used to induce a PCOS-like syndrome in animals [52] because of their ability to induce continuous estrus and cystic follicles, with morphologic characteristics resembling those observed in women. However, few studies using these treatments have demonstrated high androgen levels in blood. This was the case in a study in which neonatal female rats were treated with the endocrine disruptor bisphenol A [32], and exhibited high testosterone levels and numerous cystic and atretic follicles later in life. Possibly, acute exposure to estrogen could lead to changes in follicular enzyme activity and subsequent suppression of androgen production by theca cells. BPA is a potential agonist of endocrine estrogen, acting differentially depending on tissue estrogen receptor expression [62]. This suggests BPA could have less of an effect on regulation of hypothalamic-pituitary-ovarian axis negative feedback and, consequently, on ovarian androgen secretion. Therefore, estrogen-induced intervention may not be the optimal experimental models for study of PCOS.

Other experimental interventions using external physical stressors have been reported to induce reproductive features similar to the PCOS phenotype. Chronic cold stress may produce such changes in ovarian morphology by marked central activation of the sympathetic nerves to the ovary [42]. Such activation by cold stress is probably mediated through a regulatory mechanism on the hypothalamic-pituitary-adrenal axis by the locus coeruleus [42]. In addition, continuous light exposure could lead to changes in the estrous cycle, such as continuous estrous and cystic follicles, by altering the circadian system [41].

Letrozole, an aromatase inhibitor, was another hormonal intervention that induced high androgen levels and ovarian cysts [40] by inhibiting androgen conversion to estrogen and promoting alteration of the hypothalamic-pituitary-gonadal axis and high LH levels. In addition, by similar mechanisms, a transgenic mouse model successfully generated reproductive abnormalities by promoting recombination of the LHβ gene and hCG β-subunit [37], thus inducing chronic elevation of LH levels as well as increased testosterone and estrogen levels and cystic follicles.

Finally, although genetic rodent models cited in the present review also fail to fully replicate the reproductive phenotype of PCOS, the use of different transgenic animals may be useful to identify potential pathways involved in alterations in reproductive and endocrine aspects in these animals, which, in turn, may lead to important clinical insights into the etiology of human PCOS.

One limitation of the present systematic review is that we did not search for animal models related to the metabolic abnormalities often associated with PCOS in women. However, although insulin resistance is frequently found in PCOS, it is not considered its primary etiology. Another limitation is that we did not search for studies using other animal species than rodents and non-human primates, as these animals require no unusual laboratory facilities. A third limitation is that we did not perform meta-analysis, as the great heterogeneity in animal species and experimental procedures precluded clustering of different studies to test the efficacy of each model in producing the expected characteristics.

## Conclusions

This systematic review included 39 reports of animal models inducing two of the most characteristics features of human PCOS: hyperandrogenism and ovarian morphology changes. These studies used different experimental procedures and model organisms. Although acute estradiol administration was associated with development of cystic follicles, most of the studies were unable to demonstrate testosterone overproduction. While other interventions, mainly transgenic animals, were able to induce hyperandrogenism and cystic follicles, medium- or long-term testosterone administration in the pre- and postnatal periods performed best for induction of a PCOS-like phenotype, in rhesus macaque and rat models respectively. In rats, postnatal exposure to androgens results in reprogramming of the hypothalamic-pituitary-ovarian-axis. Thus, comparisons between different intervention models may be useful to define the timing of reproductive PCOS phenotypes in experimental animal models.

## Abbreviations

BPA: Bisphenol A
CL: corpus luteum
DHEA: Dehydroepiandrosterone
DHT: Dihydrotestosterone
E2: Estradiol
eCG: Equine chorionic gonadotropin
FSH: Follicle stimulating hormone
GnRH: Gonadotropin releasing hormone
hCG: Human chorionic gonadotropin
LH: Luteinizing hormone
MeSH: Medical subject headings
NZO: New Zealand obese mice
PCOS: Polycystic ovary syndrome
T: Testosterone
